# The effect of muscles in the treatment of lower limb lymphedema: respiratory muscles or leg muscles?

**DOI:** 10.1007/s00520-025-09436-3

**Published:** 2025-04-11

**Authors:** Ahmet Akgul, İlknur Mazi, Gamze Aydin, Mumine Yavuz, İpek Yeldan

**Affiliations:** 1 Faculty of Health Sciences, Division of Gerontology, Istanbul University-Cerrahpasa, Istanbul, Turkey; 2 Faculty of Health Sciences, Division of Physiotherapy and Rehabilitation, Istanbul Okan University, Istanbul, Turkey; 3 Faculty of Health Sciences, Division of Physiotherapy and Rehabilitation, Istanbul University-Cerrahpasa, Istanbul, Turkey

**Keywords:** Lower limb, Lymphedema, Manual lymphatic drainage, Muscle training, Respiratory

## Abstract

**Purpose:**

This study aimed to compare the effects of manual lymphatic drainage and bandaging (MLDB) combined with calf muscle exercise training (CMT) and/or inspiratory muscle training (IMT) on edema, muscle strength, functional capacity, functionality, and quality of life (QoL) in patients with secondary lower limb lymphedema (LLL).

**Method:**

A total of 76 patients (mean age: 47.06 ± 16.16 years; 84.2% female) with LLL were included in the study and randomized into four groups: MLDB alone (Group 1), MLDB + CMT (Group 2), MLDB + IMT (Group 3), and MLDB + CMT + IMT (Group 4). The training programs were administered for 30 min per day, five days per week, over three weeks. Edema was assessed using circumference measurements (CM) and tissue dielectric constant (TDC). Muscle strength was evaluated using maximum inspiratory/expiratory pressure (MIP/MEP) and a dynamometer. Functional capacity was assessed with the 6-Minute Walk Test (6MWT), functionality with the Lower Extremity Functional Scale (LEFS), and QoL with the Lymphedema Quality of Life Scale (LYMQOL).

**Results:**

In the intra-group analyses, all assessments improved in all groups, except for MIP, MEP, and gastrocnemius muscle strength in Group 1 and MIP in Group 2 (p < 0.05). In the inter-group analyses, Group 3 showed the largest effect sizes (ES) for reductions in TDC (ES: 2.34) and improvements in LYMQOL (ES: 1.74), MEP (ES: 1.46), and LEFS (ES: 1.44) (p < 0.001 for all). Group 4 had the largest ES for increases in MIP (ES: 1.42, p < 0.001). Group 2 showed the largest ES for improvements in gastrocnemius muscle strength (ES: 1.41, p < 0.001). However, there were no significant differences among the groups in CM or 6MWT results (p > 0.05).

**Conclusion:**

Compared to enhancing leg muscle strength, improving respiratory muscle function in addition to MLDB had a greater impact on reducing edema and enhancing functionality and QoL.

**Trial Registration Number:**

NCT05609526. Registration Date: 14.11.2022.

## Introductıon

Lymphedema is a chronic, progressive condition caused by abnormal development or damage to the lymphatic system, leading to the accumulation of protein-rich fluid in the interstitial tissue spaces [1, 2]. Patients with lower extremity lymphedema (LLL) commonly experience swelling, reduced joint range of motion, fatigue, infections, ulceration, sensory impairment, deformity, body asymmetry, and muscle weakness [3]. The increased volume in the affected limb due to edema, along with a sensation of heaviness, can make walking and daily activities challenging, ultimately reducing functional capacity and performance [3, 4]. The primary goals of treatment for lower limb lymphedema (LLL) are to minimize swelling, prevent complications, and restore limb functionality. Managing LLL typically requires lifelong care and may involve conservative, medical, and sometimes surgical interventions [5]. The gold standard treatment is complex decongestive physiotherapy (CDP), which includes manual lymphatic drainage and bandaging (MLDB), skin care, and exercise, all provided by trained physiotherapists [1, 6–8]. CDP has been shown to effectively reduce limb volume and edema while improving function and quality of life (QoL) in patients with lymphedema [9, 10].

Exercise activates the musculoskeletal system, increasing lymph flow and accelerating protein reabsorption [11, 12]. Additionally, deep inspiration during respiration increases tidal volume, triggering diaphragmatic movement and causing more pronounced pressure changes in the thoracic and abdominal cavities. Since these cavities house major veins and lymphatic collecting channels, deep and effective inspiration is expected to enhance lymphatic return, given the slow, rhythmic movement of larger lymphatic collectors and their anatomical positioning within intrathoracic and intra-abdominal cavities [12, 13].

The pump function of skeletal muscles, particularly the calf muscles in LLL and the respiratory muscles, supports venous return, indirectly stimulating lymph circulation and reducing edema. Studies have shown that improved respiratory and calf muscle pump function positively affects venous refilling time in patients with chronic venous insufficiency [14]. Since venous and lymphatic return work in parallel, an increase in venous return can positively impact lymphatic flow. However, most studies have focused on breast cancer-related upper limb lymphedema, reporting that different exercise training programs influence function and QoL in varying ways [15–17]. In contrast, lower-limb exercises, including hip, knee, and ankle mobility exercises, as well as active range of motion exercises that do not specifically target the calf muscles, have been reported to be effective in reducing lower-limb edema secondary to cancer [18, 19].

Although respiratory exercises play a crucial role in lymphedema management, no study has investigated the effects of inspiratory muscle training (IMT), either alone or in combination with MLDB, on secondary LLL. Additionally, evidence on the effectiveness of calf muscle strengthening training (CMT) in patients with secondary LLL remains limited [19]. Therefore, this study aimed to determine the most effective treatment combination by evaluating four different treatment options, assessing both the isolated and combined effects of these exercise training programs on the two muscle pump mechanisms in secondary LLL.

In this study, we hypothesized whether respiratory muscles or leg muscles would provide greater benefits in terms of edema reduction, muscle strength, functional capacity, functionality, and QoL. Additionally, we aimed to determine which treatment combinations could serve as viable alternatives to each other. The objective of this study is to compare the effects of MLDB combined with IMT and/or CMT on edema, respiratory and calf muscle strength, functional capacity, functionality, and QoL in patients with secondary LLL.

### Method

The study had a prospective, randomized controlled, double-blind study design. Approval was granted by the Ethics Committee of the Istanbul University-Cerrahpasa (26.12.2022–2023/10). This study was registered with ClinicalTrials.gov (NCT05609526). It was conducted in accordance with the principles of the Declaration of Helsinki. All individuals included in the study were informed about the assessments and interventions, and informed consent was obtained. The study took place at the Research and Application Center, in university hospital.

The inclusion criteria were a diagnosis of secondary lymphedema at stages 1, 2, or 3 and an age range of 18 to 75 years. Participants were excluded if they had chronic respiratory diseases, New York Heart Association (NYHA) class III and/or IV heart failure, neurological, orthopedic, or rheumatologic conditions, active infections, concurrent lipoedema with lymphedema, or primary or malignant lymphedema.

## Sample size

The power analysis for this study was conducted using G*Power 3.1.9.7. The analysis yielded an effect size of 0.40 with a 95% confidence level and 80% statistical power [20]. Based on these results, the study aimed to recruit a minimum of 76 participants, with 19 participants assigned to each group of secondary LLL.

## Randomization and blinding

The Research Randomizer website was used to assign participants to groups, and the generated numbers were placed into sealed envelopes. Groups were formed based on randomly drawn numbers [21]. Randomization was conducted confidentially to maintain blinding. A cardiovascular surgeon performed the randomization, while assessments were carried out by a physiotherapist who was blinded to group allocation. The interventions were administered by two blinded physiotherapists, and statistical analyses were conducted by a blinded researcher. All patients were randomized using concealed allocation.

**Fig. 1:**
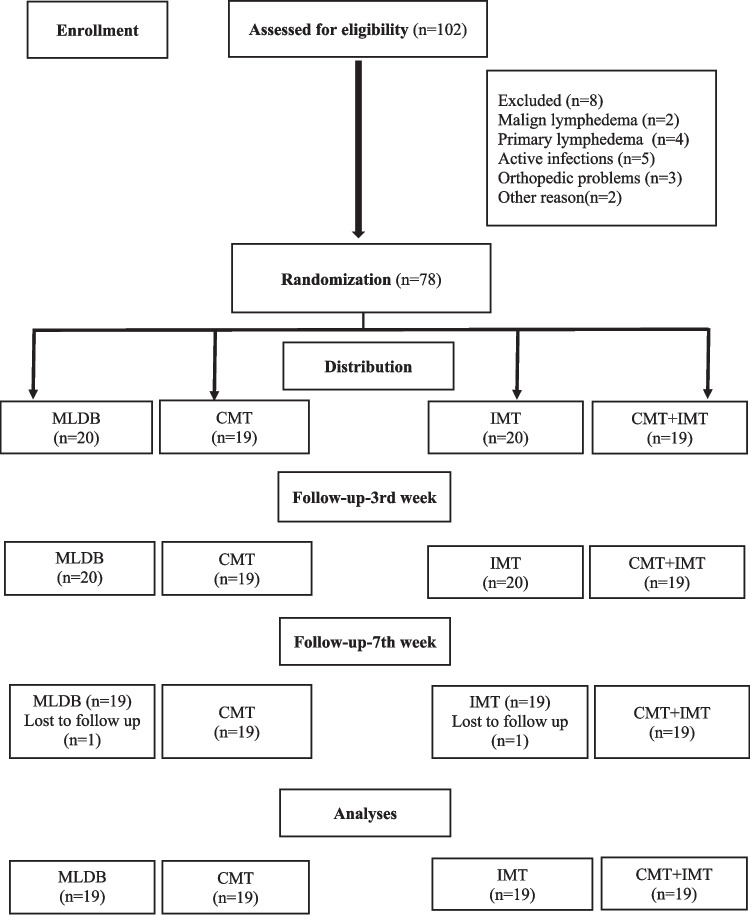
Flow Chart-CONSORT Diagram

The participants were randomized into four groups: MLDB alone (group 1), MLDB + CMT (group 2), MLDB + IMT (group 3), and MLDB + CMT + IMT (group 4). The flow diagram of patients through the trial is illustrated in Fig. 1.

## Outcome measurements

Participants'demographic and disease information (age, gender, height, body weight, stage and duration of lymphedema) was recorded.

## Assessments of edema

Leg edema was assessed by measuring the circumference of the lower extremity, from the hallux to the thigh, with marks every 10 cm using a tape measure. The Tissue Dielectric Constant (TDC) technique provides valuable information about the onset of lymphedema in its early stages and changes in subcutaneous water content. TDC was measured using a Moisture Meter-D (Delfin Technologies Ltd., Kuopio, Finland) on the distal and anterior tibia. The probe is placed in contact with the skin, and the device generates a high-frequency electromagnetic wave of 300 MHz, which is transmitted through a coaxial probe to the skin. This wave carries information about the water content of the measured tissue. The dielectric constant is a dimensionless physical quantity, and the device automatically converts the measured dielectric constant value into a tissue percentage. This percentage indicates the water content at the measurement site, with a higher percentage reflecting higher tissue water content [22].

## Assessments of muscle strength

Inspiratory and expiratory muscle strength were evaluated using the MEC Pocket-Spiro MPM 100 (Medical Electronic Construction, Brussels, Belgium), a maximum oral pressure measurement device. The assessments were conducted following the protocol published by the American Thoracic Society [23]. Maximum inspiratory intraoral pressure (MIP) was measured to assess inspiratory muscle strength, while maximum expiratory intraoral pressure (MEP) was measured to assess expiratory muscle strength.

During the test, patients were instructed to sit in a relaxed position with their upper chest and shoulders relaxed. After placing a nose clip, they were asked to tightly seal their lips around the mouthpiece of the device. For the MIP test, patients first performed a maximal expiration, after which the system was closed with a valve, and they were instructed to perform a maximal inspiration against the closed valve for at least 2 s. The procedure was reversed for the MEP test. Each measurement was conducted three times, and the mean of the three measurements was recorded.

The muscle strength of the calf muscles (gastrocnemius and soleus) was measured using the PowerTrack II Commander Muscle Testing device (JTECH Medical, Midvale, UT). Measurements were conducted three times following the manual muscle testing guidelines of Daniels and Worthingham [24], and the average values were used for statistical analysis.

Participants were positioned in a prone position with their feet hanging off the edge of the bed. Resistance was applied to the underside of the foot, just above the metatarsophalangeal joints, while participants performed plantar flexion through their full range of motion.

## Assessments of functional capacity and functionality

Functional capacity was assessed using the 6-Minute Walk Test (6MWT) following the protocol published by the American Thoracic Society. The test was conducted in a 30-m corridor, where participants were instructed to walk"as fast as possible for 6 min without running or jogging."The total distance walked within 6 min was recorded [25].

Functionality was evaluated using the Lower Extremity Functional Scale (LEFS), a questionnaire that assesses the level of difficulty experienced during 20 different daily activities. Each activity is scored from 0 to 4, with"0"indicating extreme difficulty or inability to perform the activity, and"4"indicating no difficulty. Higher scores reflect better functional capacity [26].

## Assessments of quality of life

QoL was assessed using the Lymphedema Quality of Life Scale (LYMQOL), which consists of four subscales: function, appearance, symptoms, and emotional status. Each item is rated on a scale from 1 to 4 (1 = none to 4 = very much). The score for each subscale is calculated by dividing the total score obtained by the number of items. Higher scores indicate a greater negative impact of the disease on QoL [27].

## Interventions

### MLDB

The MLDB program, which includes manual lymphatic drainage, bandaging, and skin care, was administered in the Vodder’s technique by a physiotherapist specialized in lymphedema to all groups (30 min per day, 5 days per week, for 3 weeks). MLD involves a gentle, skin-stretching massage aimed at facilitating the drainage of lymphatic fluid from the swollen limb. The drainage procedures involved positioning the patient in different postures and targeting specific anatomical regions. In the supine and prone position, drainage was performed in the cervical and abdominal regions, and for the affected lower limbs. Additionally, appropriate anastomoses were established to direct towards the axilla and unaffected inguinal region [28]. After completing MLD in all patients, the process moved on to the skin care phase. The lower limbs were moistened with a neutral pH water-based moisturizer.

The compression bandage was applied after skin care and involved the use of stockinette, finger bandages, cotton, sponges, and short stretch bandages applied from the toes to the groin. In routine conditions, patients were asked to protect their bandages on until the next session. They were also provided with instructions on how to consult with us if they experienced symptoms such as bruising or numbness in their toes, and how to safely remove the bandages if needed.

### CMT

Progressive resistance exercises for calf muscles (gastrocnemius and soleus) performed by physiotherapists, using elastic bands (red–green–blue bands, starting from low to high resistance), single and double leg heel/toe raises with body weight, standing still exercises, ankle pumping, mini squat exercises were implemented, increasing sets and repetitions, for 30 min per day, 5 days a week, over 3 weeks [29, 30]. The one repetition of maximum for muscle strength was measured again at the beginning of each week to determine the new workload.

### IMT

IMT was applied parallel to the"threshold inspiratory resistance training"protocol by physiotherapists (30 min per day, 5 days per week, for 3 weeks) [31]. Training began at 30% of the MIP using the'Philips Threshold IMT'device. The MIP value was measured again at the beginning of each week to determine the new workload. During the training, patients were instructed to sit with their upper chest and shoulders relaxed, and after wearing a nose clip, they were asked to tightly seal their lips around the mouthpiece of the device. In this position, they performed 10 respiratory cycles with the device, then removed it from the mouth and rested for 4–5 breaths. They were instructed to continue this cycle for 15 min × 2 times, totally 30 min.

## Statistical analysis

The statistical analysis of the data obtained from using the Statistical Package for the Social Sciences (SPSS) Version 22.0 (SPSS Inc., Chicago, IL, USA). The normality of the data was analyzed using the Shapiro–Wilk test. The variables were described with mean (M), standard deviation (SD). In intra group analysis the Friedman test was used to compare the measurements. The Kruskal–Wallis test were used for the inter-group analyses. The within-group effect size (ES) were calculated using the formula (D)/(standard deviation of the pretreatment evaluation). An ES of 0.81 was classified as large, an ES of 0.80 to 0.51 as moderate, and an ES of 0.50 to 0.21 as small [32]. p < 0.05 was considered statistically significant.

## Results

One hundred two patients were screened for in terms of inclusion criteria. Of these, 24 did not meet the inclusion criteria (excluded (n = 8), malign lymphedema (n = 2), primary lymphedema (n = 4), active infections (n = 5), orthopedic problems (n = 3), other reason (n = 2)), resulting in a total of 78 patients who were included in the study. One participant in the group 1 and one of the group 3 participants discontinued treatment (Fig. 1). Therefore, 76 patients completed the treatments and they were analyzed at the end of the treatment.

The demographic and clinical datas of the participants were similar in all groups (p > 0.05) (Table 1).

**Table 1:** Demographic and clinical features

Variables	G1	G2	G3	G4	P value
**Age, years**	53.89 ± 16.88	40.84 ± 16.47	48.15 ± 16.10	45.36 ± 16.10	0.087^a^
**BMI, kg/m**^**2**^	29.10 ± 5.25	29.17 ± 6.89	28.03 ± 5.36	29.63 ± 8.41	0.897^a^
**Lymphedema duration, years**	10.84 ± 7.05	8.89 ± 4.81	7.26 ± 6.38	8.57 ± 7.20	0.243^b^
**Gender**					
Female	16 (84.2)	15 (78.9)	17 (89.5)	16 (84.2)	0.851^c^
Male	3 (15.8)	4 (21.1)	2 (10.5)	3 (15.8)	
**Stages of Lymphedema**					
Stage 1	3 (15.8)	2 (10.5)	4 (21.1)	4 (21.1)	0.920^c^
Stage 2	8 (42.1)	10 (52.6)	10 (52.6)	8 (42.1)	
Stage 3	8 (42.1)	7 (36.8)	5 (26.3)	7 (36.8)	
**Etiology of lymphedema**					0.813^c^
Pelvic lymph node dissection after gynaecological surgery	12 (63.2)	9 (47.4)	13 (68.4)	13 (68.4)	
Prostate cancer surgery pelvic lymph node dissection	0	1 (5.3)	0	1 (5.3)	
Lymph node biopsy	1 (5.3)	0	1 (5.3)	0	
Phlebolymphedema	2 (10.5)	4 (21.1)	1 (5.3)	1 (5.3)	
Post-traumatic lymphoedema	1 (5.3)	1 (5.3)	3 (15.8)	1 (5.3)	
Lymphedema after pregnancy	1 (5.3)	1 (5.3)	1 (5.3)	1 (5.3)	
Others	2 (10.5)	3 (15.8)	0	2 (10.5)	
**Affected side**					
R	9 (47.4)	12 (63.2)	11 (57.9)	7 (36.8)	0.375^c^
L	10 (52.6)	7 (36.8)	8 (42.1)	12 (63.2)	
**Dominant side**					
R	13 (68.4)	14 (73.7)	15 (78.9)	16 (84.2)	0.692^c^
L	6 (31.6)	5 (26.3)	4 (21.1)	3 (15.8)	
**Previous treatments**					
None	3 (15.8)	4 (21.1)	3 (15.8)	7 (36.8)	0.316^c^
CDT	4 (21.1)	5 (26.3)	3 (15.8)	4 (21.1)	
MLD	2 (10.5)	1 (5.3)	3 (15.8)	1 (5.3)	
Compression garment/clothing	2 (10.5)	2 (10.5)	–	2 (10.5)	
Compression stockings	4 (21.1)	2 (10.5)	2 (10.5)	2 (10.5)	
Pneumatic Compression	1 (5.3)	3 (15.8)	2 (10.5)	2 (10.5)	
Classic Massage	–	1 (5.3)	2 (10.5)	–	
Pharmacological treatments	3 (15.8)	–	2 (10.5)	1 (5.3)	
Surgery	–	1 (5.3)	2 (10.5)	–	

*BMI* Body mass index, *CDT* Complex degongestive therapy, *R* right, *L* left; *G1* group 1, manual lymphatic drainage and bandaging (MLDB) alone; *G2* group 2, MLDB + CMT; *G3* group 3, MLDB + IMT; G4: group 4, MLDB + CMT + IMT. Data presented as mean ± standard deviation or number (%). a: One-way analysis of variance. b: Kruskal–Wallis test. c: Chi-square test

In intra-group analysis demonstrated that edema of the affected lower extremity decreased in all groups (p < 0.001), however there were no significant differences between groups (p > 0.05) (Table 2). Additionally, reductions in TDC values were detected within all groups in intra-group analysis (p:0.002, p:0.000, p:0.000, p:0.000, respectively), with the largest decrease observed in Group 3 when examining ES (p:0.000, ES: 2.34) (Table 3).

**Table 2:** Comparison of parameters related with edema (circumference measurements, tissue dielectric constant, perception of leg heaviness)

Edema	G1			G2			G3			G4		
	**BT**	**AT**	**A1M**	**BT**	**AT**	**A1M**	**BT**	**AT**	**A1M**	**BT**	**AT**	**A1M**
**10. cm**	24.36 ± 2.22	22.63 ± 1.92	22.52 ± 1.86	24.97 ± 3.02	22.89 ± 2.70	22.68 ± 2.51	24.34 ± 1.57	23.02 ± 2.01	23.02 ± 2.01	25.05 ± 3.21	23.13 ± 2.27	22.39 ± 1.94
**20. cm**	29.26 ± 4.13	26.34 ± 3.36	25.55 ± 3.10	32.83 ± 10.96	26.92 ± 5.08	26.26 ± 5.01	27.57 ± 4.22	25.52 ± 4.53	25.26 ± 4.57	31.34 ± 7.55	26.47 ± 4.24	25.34 ± 4.16
**30. cm**	36.55 ± 6.40	31.78 ± 4.23	31.00 ± 4.38	39.36 ± 11.65	33.07 ± 7.50	32.26 ± 7.63	33.86 ± 5.67	30.63 ± 5.15	30.68 ± 5.59	38.81 ± 8.72	32.92 ± 5.54	31.63 ± 5.17
**40. cm**	41.15 ± 6.29	37.26 ± 5.07	36.63 ± 4.91	43.73 ± 10.14	37.89 ± 7.76	36.78 ± 7.56	40.50 ± 6.04	38.05 ± 5.42	37.47 ± 5.16	42.44 ± 8.82	37.00 ± 6.75	35.92 ± 6.59
**50. cm**	42.94 ± 8.65	38.26 ± 6.89	38.10 ± 6.83	42.47 ± 9.10	38.86 ± 7.74	38.10 ± 7.53	41.76 ± 7.83	39.57 ± 7.20	38.78 ± 6.93	43.13 ± 10.86	39.00 ± 9.25	37.86 ± 8.60
**Edema**		**Within-group P value**^**a**^** (ES)**							**Between group P value**^**b**^			
		**G1**	**G2**	**G3**	**G4**				**BT**	**AT**		**A1M**
**10. cm**		**0.000**(− 0.828)	**0.000**(− 0.756)	**0.000**(− 0.836))	**0.000**(− 0.825)				0.973	0.921		0.819
**20. cm**		**0.000**(− 0.896)	**0.000**(− 0.598)	**0.000**(− 0.547)	**0.000**(− 0.794)				0.419	0.641		0.863
**30. cm**		**0.000**(− 0.866)	**0.000**(− 0.609)	**0.000**(− 0.561)	**0.000**(− 0.823)				0.320	0.416		0.882
**40. cm**		**0.000**(− 0.719)	**0.000**(− 0.684)	**0.000**(− 0.500)	**0.000**(− 0.739)				0.798	0.778		0.612
**50. cm**		**0.000**(− 0.559)	**0.000**(− 0.479)	**0.000**(− 0.379)	**0.000**(− 0.484)				0.886	0.949		0.895

*AT* After treatment, *BT* before treatment, *A1M* one month after treatment, *ES* effect size, *G1* group 1, *G2* group 2, *G3* group 3, *G4* group 4 Data presented as mean ± standard or mean (95% confidence interval). Boldface *P* values represent statistical significance. a: Friedman test. b: Kruskal wallis test

**Table 3:** Comparison of muscle strength, functional capacity, functionality and quality of life

Assessments	G1				G2				G3					G4				
	**BT**	**AT**		**A1M**	**BT**	**AT**		**A1M**	**BT**		**AT**	**A1M**		**BT**		**AT**		**A1M**
**MIP**	69.52 ± 7.85	69.84 ± 7.86		69.84 ± 7.86	67.05 ± 8.28	67.57 ± 8.31		66.84 ± 8.33	67.78 ± 8.11		74.63 ± 6.54	78.10 ± 6.62		66.31 ± 9.61		75.31 ± 9.01		80.00 ± 7.84
**MEP**	58.94 ± 7.54	59.47 ± 7.53		59.47 ± 7.53	56.89 ± 4.70	57.84 ± 5.26		58.10 ± 5.74	60.52 ± 5.52		65.84 ± 5.81	68.63 ± 5.33		59.94 ± 7.68		66.31 ± 7.21		68.68 ± 7.19
**GS**	264.36 ± 23.77	253.10 ± 39.42		253.63 ± 39.58	269.10 ± 24.71	297.21 ± 37.42		304.10 ± 36.21	266.31 ± 27.28		272.94 ± 41.27	272.94 ± 41.27		277.15 ± 33.57		307.89 ± 49.78		315.47 ± 49.89
**6-MWT**	512.10 ± 88.73	521.68 ± 85.21		524.89 ± 83.41	514.78 ± 91.10	523.84 ± 87.79		528.05 ± 87.99	471.89 ± 94.05		492.47 ± 95.07	504.57 ± 96.57		436.84 ± 90.67		468.21 ± 83.16		474.57 ± 82.81
**LEFS**	41.78 ± 12.22	46.94 ± 11.18		48.52 ± 10.92	45.36 ± 12.65	51.84 ± 10.97		53.89 ± 9.67	34.68 ± 10.30		45.63 ± 9.01	49.57 ± 8.66		40.73 ± 12.77		53.26 ± 10.00		57.89 ± 8.28
**LYMQOL**	45.84 ± 16.65	31.73 ± 12.57		30.73 ± 12.61	52.00 ± 11.42	44.21 ± 10.63		38.21 ± 12.21	52.78 ± 12.27		36.21 ± 7.44	31.31 ± 9.55		60.42 ± 17.86		45.42 ± 15.41		34.89 ± 13.11
**TDC**	51.73 ± 4.34	50.57 ± 5.05		50.57 ± 5.05	49.31 ± 3.84	43.52 ± 3.62		42.05 ± 3.83	50.05 ± 4.49		43.68 ± 4.58	39.52 ± 5.25		48.68 ± 8.74		39.26 ± 9.01		36.84 ± 9.20
**Assessments**		**Within-group P value**^**a**^** (ES)**											**Between group P value**^**b**^					
		**G1**	**G2**				**G3**			**G4**			**BT**		**AT**		**A1M**	
**MIP**		0.319(0.04)	0.120(–0.02)				**0.000**(1.27)			**0.000**(1.42)			0.775		**0.011**		**0.000**	
**MEP**		0.076(0.06)	**0.001**(0.25)				**0.000**(1.46)			**0.000**(1.13)			0.156		**0.000**		**0.000**	
**GS**		0.368(–0.45)	**0.000**(1.41)				**0.000**(0.24)			**0.000**(1.14)			0.196		**0.000**		**0.000**	
**6-MWT**		**0.000**(0.14)	**0.000**(0.14)				**0.000**(0.31)			**0.000**(0.41)			0.052		0.120		0.145	
**LEFS**		**0.000**(0.55)	**0.000**(0.67)				**0.000**(1.44)			**0.000**(1.34)			0.051		**0.044**		**0.005**	
**LYMQOL**		**0.000**(–0.90)	**0.000**(–1.20)				**0.000**(–1.74)			**0.000**(–1.42)			0.059		**0.002**		0.165	
**TDC**		**0.002**(–0.26)	**0.000**(–1.88)				**0.000**(–2.34)			**0.000**(–1.35)			0.308		**0.000**		**0.000**	

*AT* After treatment, *BT* before treatment, *A1M* one month after treatment, *ES* effect size, *MIP* maximum inspiratory intraoral pressure, *MEP* maximum ekspiratory intraoral pressure, *GS* gastrocnemius-soleus muscle strength, *6-MWT* 6-min walk test, *LEFS* lower extremity functional scal, *LYMQOL* lymphedema quality of life scale, *G1* group 1, *G2* group 2, *G3* group 3, *G4 * group 4. Data presented as mean ± standard or mean (95% confidence interval). Boldface P values represent statistical significance. a: Friedman test. b:Kruskal wallis test

The intra-group evaluation of MIP value, both group 3 and 4 showed increase (p:0.000, p:0.000, respectively). On the other hand, MEP value increased group 2, 3 and, 4 (p:0.001, p:0.000, p:0.000, respectively). In inter-group analyses, it was observed that the MIP value had a large ES in Group 4 (p:0.000, ES:1.42), while the MEP value had large ES in Group 3 (p:0.000, ES:1.46) (Table 3). In intragroup analyses, gastrocnemius muscle strength increased in groups 2, 3, and 4 (p:0.000, p:0.000, p:0.000, respectively). In intergroup analyses, gastrocnemius muscle strength had the largest ES in group 2 (p:0.000, ES: 1.41) (Table 3).

The intra-group assessment of the 6MWT were improved in all groups (p:0.000, p:0.000, p:0.000, p:0.000, respectively). However, no statistically significant difference was found in the 6MWT among the groups(p > 0.05) (Table 3). In addition, LEFS and LYMQOL scores significantly improved in all groups (p:0.000, p:0.000, p:0.000, p:0.000, respectively) in intra-group analyses and they had the largest ES in group 3 (ES:1.44, ES:1.74, respectively) (Table 3).

## Discussion

This study investigated the effectiveness of four different isolated and/or combined treatments, administered 5 days a week, on edema, respiratory and calf muscle strength, functional capacity, functionality, and QoL in patients with secondary LLL. It was shown that MLDB + IMT was the most effective combination of treatments for edema, expiratory muscle strength, functionality, and QoL; MLDB + CMT was the most effective for gastrocnemius and soleus muscle strength; MLDB + CMT + IMT was the most effective for inspiratory muscle strength in patients with secondary LLL. No lymphedema exacerbation or adverse effects were recorded during the study.

CDP is the primary conservative treatment strategy applicable to all patients with lymphedema. Kim et al. [9] reported a reduction in excess limb volume from 55.92% at baseline to 31.56% after one month of CDP, along with improved QoL in patients with unilateral lower-limb lymphedema following gynecological cancer treatment. Similarly, Soares et al. [10] demonstrated a significant reduction in lymphedema volume and improved QoL in lymphatic filariasis patients after undergoing CDP twice a week for ten weeks. However, no improvement was observed in lower-extremity functionality, as assessed by the Timed Up and Go test. Wu et al. [33] found that combining CDP with hip exercises for early prevention reduced the incidence of LLL, improved QoL, and decreased fatigue. This study demonstrated that MLDB alone led to improvements in edema, functional capacity, functionality, and QoL. However, the effect size of MLDB alone on these parameters was small to moderate, except for QoL. The reduction in edema and subcutaneous water content in the leg may have decreased the sensation of heaviness, facilitating more active use of the leg in daily life. This, in turn, may have contributed to an increased walking distance, improved functionality, and a positive impact on QoL.

This study demonstrated the effectiveness of CMT in addition to MLDB in reducing edema, improving gastrocnemius-soleus muscle strength, and enhancing QoL with a large effect size (ES), while functionality improved with a moderate ES. Notably, CMT was the most effective intervention for increasing gastrocnemius-soleus muscle strength compared to other treatment combinations in secondary LLL. Strengthening the calf muscles may enhance the efficiency of the muscle pump mechanism. Fukushima et al. [11] reported that lower-limb volume significantly decreased after high-load active exercise combined with compression therapy compared to compression therapy alone. Their exercise program included cycling on a bicycle ergometer, which is known to stimulate the calf muscle pump mechanism. Similarly, Zhang et al. [18] investigated resistance training of the lower limbs with an elastic bandage in a standing position for patients with LLL after pelvic lymphadenectomy for cervical cancer. They found that resistance training effectively prevented lymphedema; however, the specific muscles targeted and the training protocol were not detailed. Katz et al. [19] examined the effects of resistance exercises, including leg press, leg extension, leg curl, hip flexion, leg abduction, prone straight leg lifts, and calf raises, performed twice a week under supervision for two months, followed by three months of unsupervised training in patients with secondary LLL. They reported no clinically significant worsening in total leg volume. Although their study had a small sample size (n = 10), long-term resistance exercises for lower extremity muscles were effective in preventing edema. In the present study, the reduction in edema may have contributed to increased ankle mobility, improved calf muscle strength, and enhanced functional activities. These factors could facilitate venous return, indirectly stimulate lymphatic circulation, and ultimately help reduce edema.

The combined application of IMT with MLDB resulted in increased inspiratory and expiratory muscle strength. This improvement may be attributed to enhancements in respiratory patterns and intra-abdominal and intrathoracic pressure regulation, which likely facilitated lymphatic flow. Additionally, IMT combined with MLDB was the most effective intervention for reducing subcutaneous water content, which positively influenced both functionality and perceived QoL. The reduction in leg volume significantly improved lower extremity functionality, yielding a larger effect size compared to other interventions. Notably, there are no prior studies examining the effectiveness of IMT in patients with LLL. Aydın et al. [14] reported that an 8-week IMT program resulted in increased respiratory muscle strength and improved venous refill time, as assessed by photoplethysmography, in patients with chronic venous insufficiency. In a separate study, Aydın et al. [34] demonstrated a positive association between MIP and venous refill time, indicating that stronger respiratory muscles contribute to improved venous return. Since venous and lymphatic return function in parallel, an increase in venous return likely enhances lymphatic flow as well. Similarly, Abreu et al. [35] found significant impairments in lung function and respiratory muscle strength in post-mastectomy patients, reporting reductions in MIP, MEP, and FEV1. Espino-López et al. [36] observed mean MIP and MEP values ranging from 43.9 ± 7.9 to 65.0 ± 9.1 cmH2O and 36.0 ± 7.6 to 48.6 ± 7.5 cmH2O, respectively, with expected percentage values of 41% and 33%. Kutlu et al. [37] revealed that after breast cancer treatment, 76% of female patients developed lymphedema, and their functional exercise capacity, MIP, and MEP were below expected levels. Their study also reported decreased respiratory muscle strength, a reduced FEV1/FVC ratio, and diminished walking distance in patients with upper limb lymphedema. A common finding across these studies is that upper limb lymphedema is associated with impaired respiratory function and a weakened respiratory pump. Similarly, this study found lower baseline MIP and MEP values (ranging from 66.31 ± 9.61 to 69.52 ± 7.85 for MIP and 56.89 ± 4.70 to 60.52 ± 5.52 for MEP). However, our primary aim was to evaluate the effects of IMT on edema and functional outcomes rather than solely on respiratory function.

The IMT and CMT in which the patient actively participates in managing lymphedema symptoms, positively impacted the evaluation outcomes compared to MLDB alone, where the patient plays a passive role. Active involvement of the patient in the treatment may have increased their adherence to and motivation for the therapy. It is recommended that this aspect be evaluated in future studies. In addition, the difficulty of applying CMT for patients and the fact that IMT is easier to apply for patients of all severities, along with its observed effectiveness in results, suggest that IMT should be included as a routine treatment for lymphedema patients.

A key strength of this study is its structured design, which involved applying four different treatment combinations five days a week over three weeks. This approach allowed for a comprehensive evaluation of the effectiveness of both isolated and combined interventions targeting respiratory and leg muscles. Furthermore, the study utilized objective methods to assess improvements in edema and muscle strength, enhancing the reliability of the findings.

However, a limitation of the study is that it included only patients with secondary lymphedema. As a result, the findings may not be fully generalizable to all individuals with lymphedema.

## Conclusion

This study is the first to examine the effects of both respiratory and leg muscle activity on edema, muscle strength, functionality, and quality of life in patients with LLL. The findings suggest that, in addition to MLDB, improving respiratory muscle efficiency has a greater impact on edema reduction, functionality, and quality of life compared to strengthening leg muscles.

## Data Availability

No datasets were generated or analysed during the current study.
